# Supplementary material for: High Flexibility of RNaseH2 Catalytic Activity with Respect to Non-Canonical DNA Structures
Source: Int J Mol Sci. 2021 May 14;22(10):5201. doi: 10.3390/ijms22105201 (PMC8155979; doi:10.3390/ijms22105201)
Supplement: Supplementary file 1 [file ijms-22-05201-s001.zip › ijms-1215578-supplementary.pdf]

Supplementary Materials

High Flexibility of RNaseH2 Catalytic Activity with Respect to Non-Canonical DNA Structures

Maria Dede [1,†], Silvia Napolitano [1,†,‡], Anna Melati [1], Valentina Pirota [2], Giovanni Maga [1] and Emmanuele Crespan [1,*]

1 Institute of Molecular Genetics IGM-CNR "Luigi Luca Cavalli-Sforza", via Abbiategrasso 207, I-27100 Pavia, Italy; maria.dede01@universitadipavia.it (M.D.); silvia.napolitano@mol.biol.ethz.ch (S.N.); anna.melati01@universitadipavia.it (A.M.); giovanni.maga@igm.cnr.it (G.M.)
2 Chemistry Department, University of Pavia, Via Taramelli 12, I-27100 Pavia, Italy; valentina.pirota@unipv.it
* Correspondence: crespan@igm.cnr.it
† These authors contributed equally to this work.
‡ Present address: Institute of Molecular Biology and Biophysics, ETH, CH-8093 Zurich, Switzerland

Contents: Supplementary Table S1; Supplementary Methods; Supplementary Results; and Supplementary Figures S1–S7; Supplementary Bibliography

Table S1. Substrates and oligonucleotides sequences.

Substrate	Oligo	Sequence
S1	#1	5′Fam-GAGTTTGAGTGGAGGTGTG(rC)GTAGGGTAGTATTGGTGGATA-3′
	#S1	5′-TGAGGAGTTGGTTACACACCTCCACTCAAACTC-3′
S2	#1	5′Fam-GAGTTTGAGTGGAGGTGTG(rC)GTAGGGTAGTATTGGTGGATA-3′
	#S2	5′-TATCCACCAATACTACCCTACACACACCTCCACTCAAACTC-3′
S3	#S3	5′Fam-GAGAGAAGATGGAAGTGAGGAA(rC)TGAGGGTGGGTAGGGTGGGTAA-3′
	#18	5′-AAAAAAAAAAAAAAAAAAAAAGTTCCTCACTTCCATCTTCTCTC-3′
S4	#S4	5′Fam-TGAGGGTGGGTAGGGTGGGTAA(rC)GAGAGAAGATGGAAGTGAGGAA-3′
		5′-TTCCTCACTTCCATCTTCTCTCGAAAAAAAAAAAAAAAAAAAAA-3′

Supplementary methods.

RNase H2 Relative Activity. Relative activity was calculated posing, for each independent experiment and for each time point considered, the activity obtained for substrate1 as 100% and calculating the relative activity on the compared substrate.

CD Spectra. All oligonucleotides were diluted from stocks to final concentration (5 μM for G4 structure and 10 μM for duplex and hairpin) in Hepes-LiOH buffer (30 mM, pH 7.4) containing 2 mM $MgCl_2$, 50 mM NH_4Ac and 100 mM KCl. G4 controls were prepared substituting KCl with 100 mM LiCl. The solutions were annealed heating at 95°C for 5 min and gradually cooling to room temperature overnight.

CD spectra were recorded on Jasco model J-1500 spectropolarimeter at 310 K using a quartz cell of 1 mm optical length, an instrument scanning speed of 50 nm/min with a response of 2 s over a wavelength range of 215-340 nm with a 1 nm sampling interval. The reported spectra of each sample represent the average of 3 scans and are baseline-corrected for signal contributions due to the buffer mixture. Observed ellipticities (in millidegrees) were converted into Molecular Ellipticity in function of sample concentrations, path length and number of residues that composed the oligonucleotides analysed. For the CD-melting experiments of G4 structures, the ellipticity was recorded at 264 nm ever 0.5°C with a temperature scan rate of 5 °C/min in the range 20–95 °C.

Supplementary results.

Evaluation of G4 formation by CD-spectra and Tm analysis. In presence of K^+ ions, oligonucleotides #**17** exhibited a maximum around 264 nm and a minimum around 243 nm, which are typical signatures of a parallel G4 topology (Supp. Figure S5 A, bold black lines) in which all the G-tracts proceed in the same direction [1]. Moreover, the additional characteristic positive peak at below 220 nm also confirms G4 folding, differentiating the signals from which of A-form DNA (which presents a negative signal in the same region) [2].

In absence of KCl, the dichroic signal of Oligo #**17** (Figure S5 A, dashed grey line) is particularly low, showing a maximum at 260 nm, a minimum at 241 nm and a positive footprint at below 220 nm. This is consistent with the presence of NH_4^+ and Mg^{2+} cations in solution, able to partially stabilize G4 structures [3].

On the contrary, the CD spectra of #**S3** and #**S4** annealed in presence of Li^+ ions (Figure S5 B and C respectively, dashed lines) resulted comparable to which obtained in presence of K^+, even if overall less intense. These results are consistent with reported stability of myc (pu22) structure [4]. Nonetheless, melting experiments on these oligonucleotides revealed that mid-transition temperatures (Tm) in the presence of 100 mM of K^+ is higher than 95°C for both the sequences while, in the complete absence of potassium cation, Tm values were 49.8 ± 0.8 °C and 50.7 ± 0.4 °C for 5′-G4-myc and 3′-G4-myc respectively. In reaction conditions and in the presence of partially annealed strand it reasonable that, in the absence of K^+, Substrates #S3 and #S4 did not formed stable G4 structures, allowing RNase H2 activity (Figure S6A, lanes 8-12, and S6B, lane3).

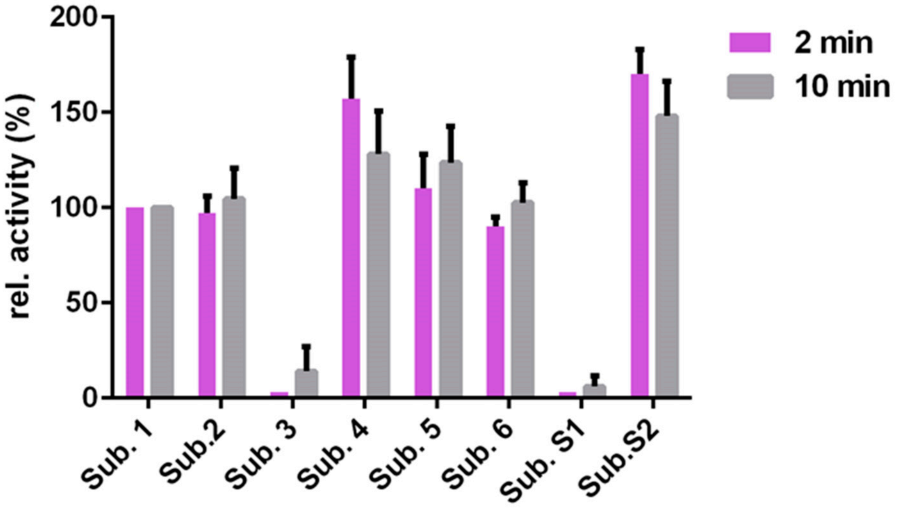

Figure S1. Comparison of RNase H2 activity on substrates showed in Figure 1. The activity of RNase H2 on substrates **2**, **3**, **4**, **5**, **6**, and **S1** was compared with activity on substrate **1** at two time points (2 and 10 minutes). Values are the mean of three independent experiments. Error bars represent ± S.D.

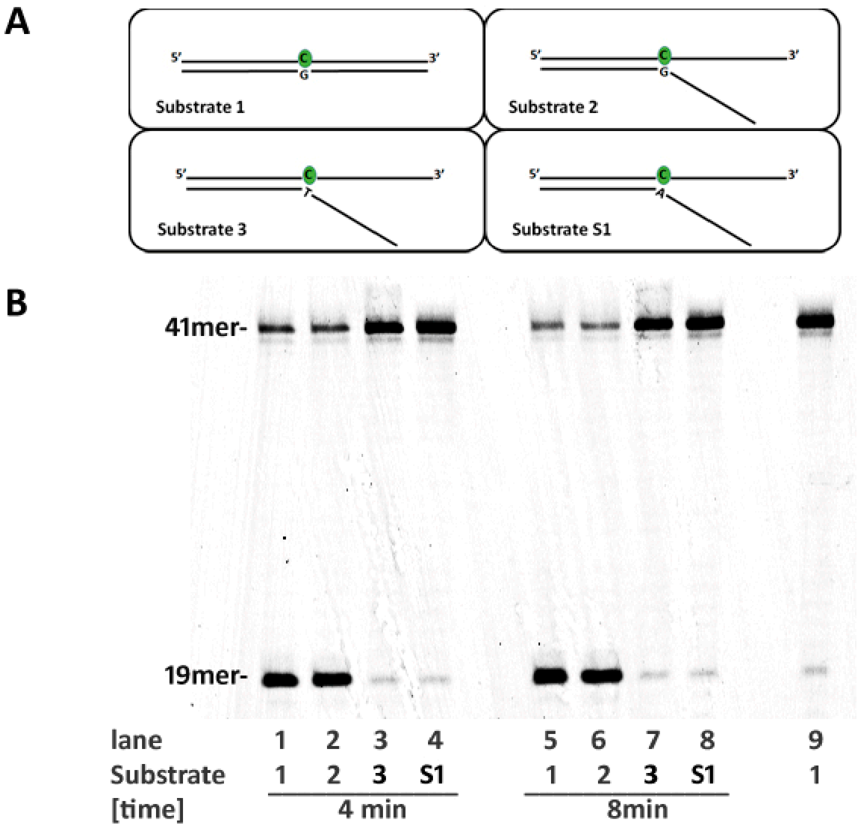

Figure S2. Comparison of RNase H2 activity on substrates presenting a flap structure flanking the 3′ of correctly paired or mispaired ribonucleotide. **A**. Schematic representation of substrates used. (**B**) Enzyme activity was compared at two time points (4 and 8 minutes) between substrate 1, 2, 3, and S1.

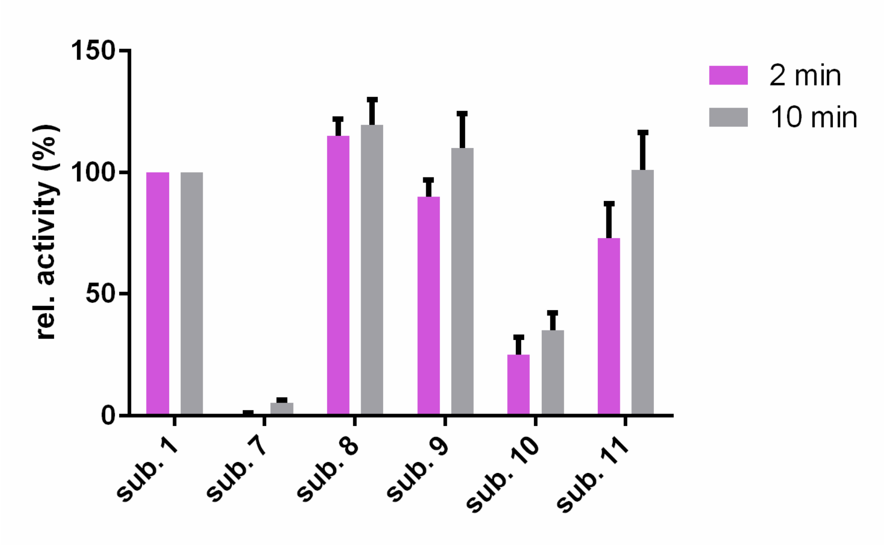

Figure S3. Comparison of RNase H2 activity on substrates with different length of double stranded structure flanking the 5′ side of the embedded ribonucleotide. The activity of RNase H2 on substrates **7**, **8**, **9**, **10**, and **11** was compared with activity on substrate **1** at two time points (2 and 10 minutes). Values are the mean of three independent experiments. Error bars represent ± S.D.

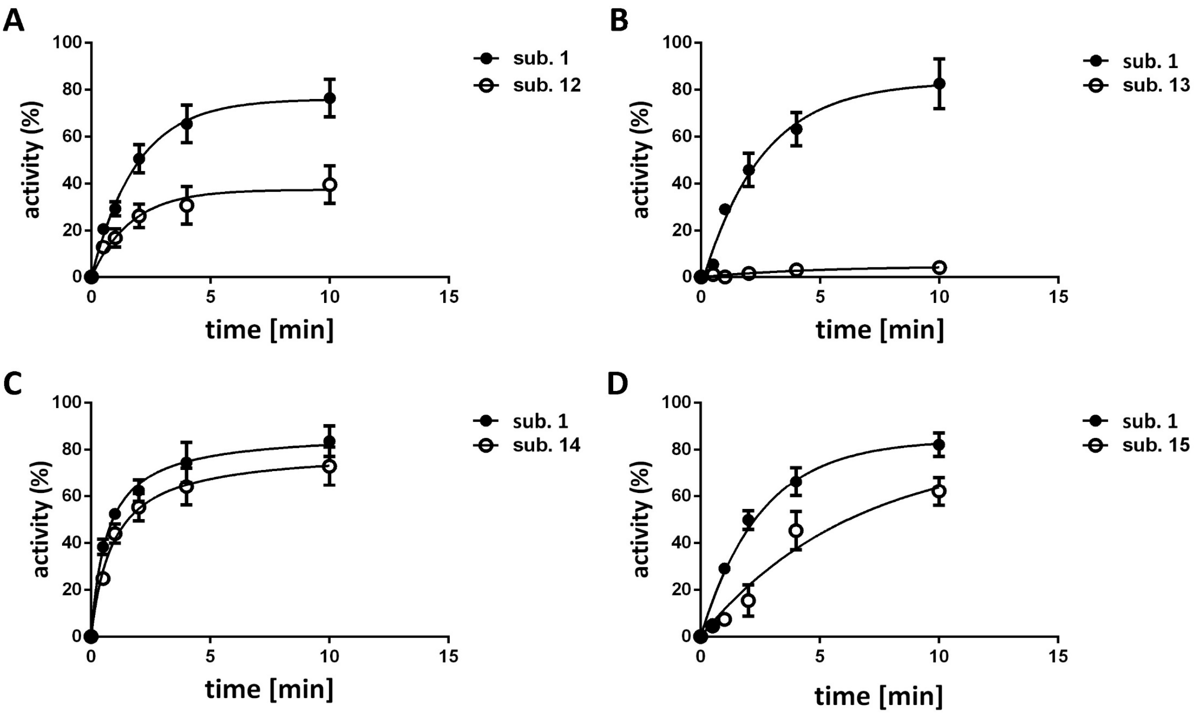

Figure S4. Quantification of RNase H2 activity on substrates containing mismatched base pairs. Values are the mean of at least three independent experiments. Error bars represent ± S.D.

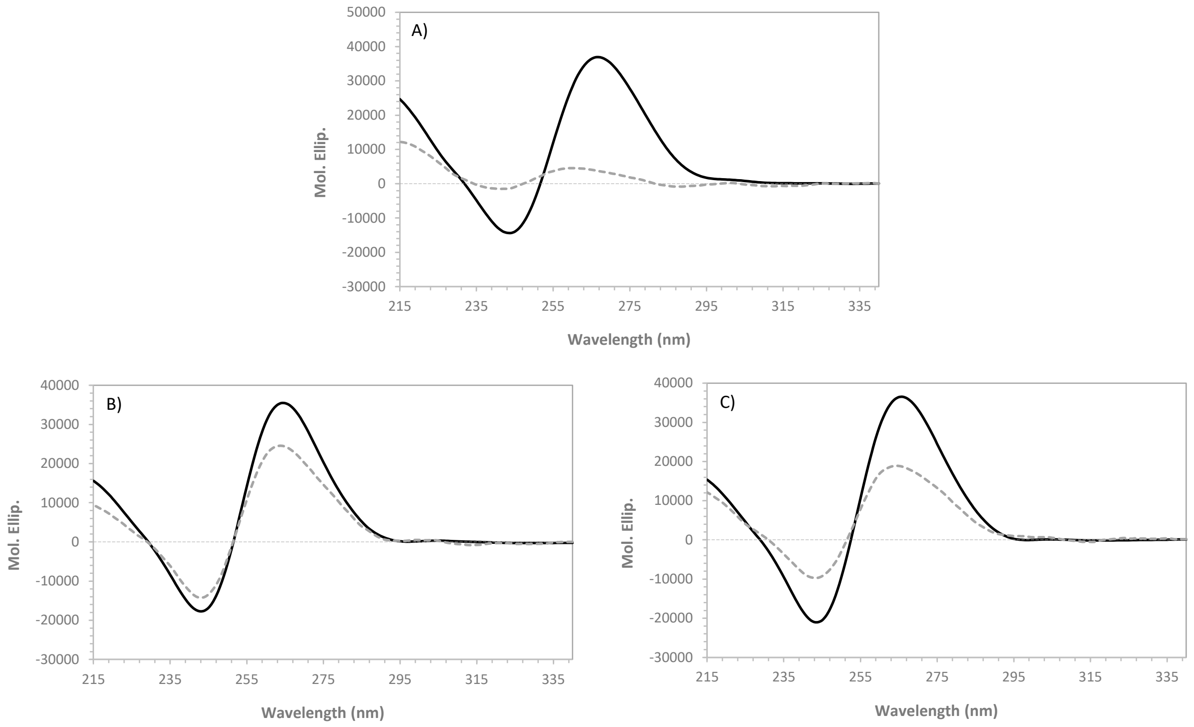

Figure S5. CD spectra of 5 μM (**A**) oligo #**17**, (**B**) oligo #**S4**, and (**C**) oligo #**S3** in Hepes-LiOH buffer (30 mM, pH 7.4) containing 2 mM $MgCl_2$, 50 mM NH_4Ac and LiCl (dashed grey lines) or KCl (bold black lines) both at 100 mM concentration.

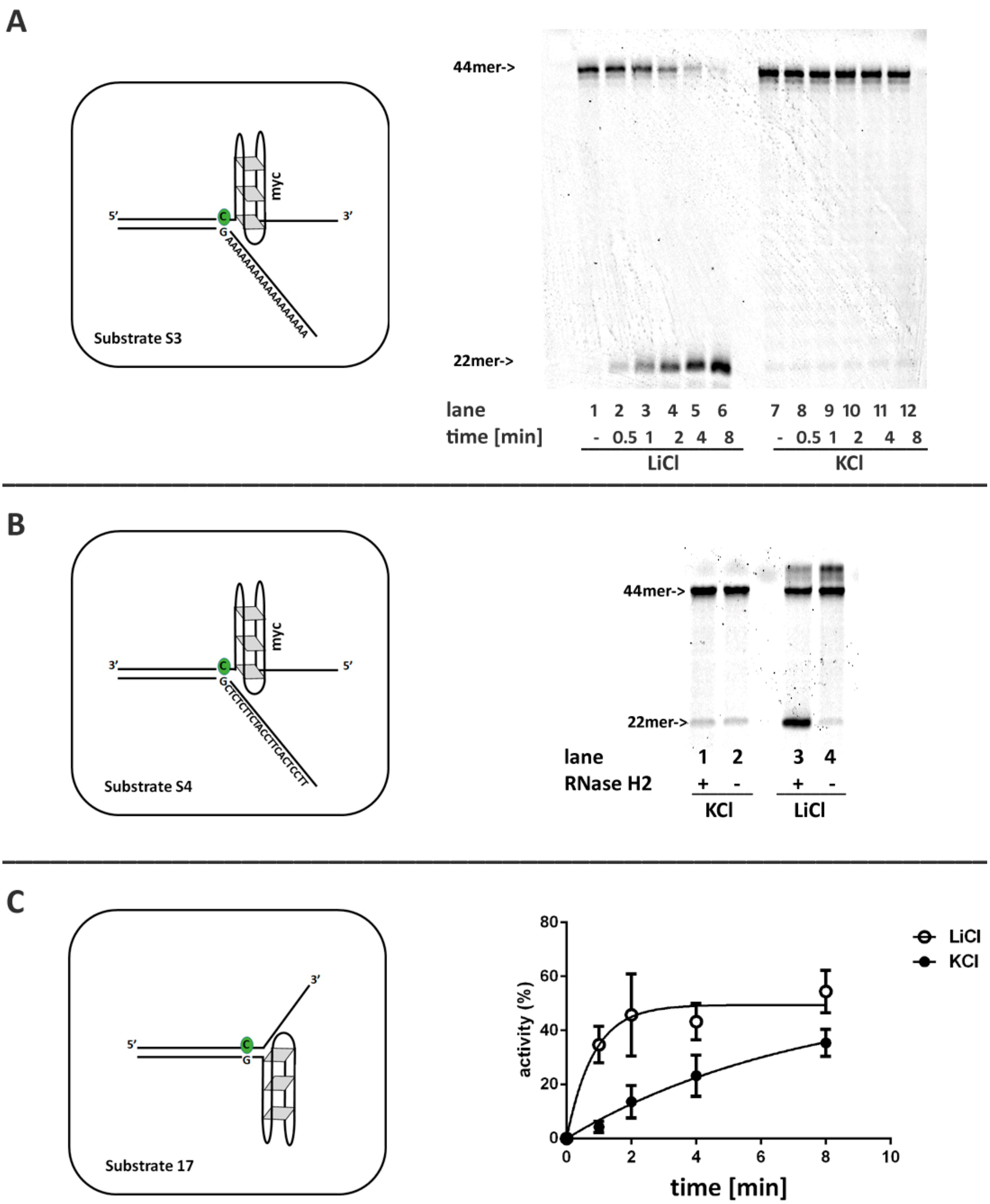

Figure S6. RNase H2 activity on Substrate presenting G4 structures. On the left are schematically represented the substrate used annealed in the presence of KCl. (**A**) Time course reactions were performed as described in materials and methods using 20 nM of substrate **S3** annealed in the presence of of LiCl (not allowing G4 formation) or KCl (allowing G4 formation). Unprocessed substrates and reaction products are indicated at the left of each panel (44mer and 22mer respectively). (**B**) RNase H2 reactions were performed as described in materials and methods and conducted for 8 minutes. Unprocessed substrates and reaction products are indicated at the left of each panel (44mer and 22mer respectively). (**C**) Quantification of RNase H2 activity on substrate **17** annealed in the presence of LiCl (not allowing G4 formation) or KCl (allowing G4 formation). Values are the mean of at least three independent experiments. Error bars represent ± S.D.

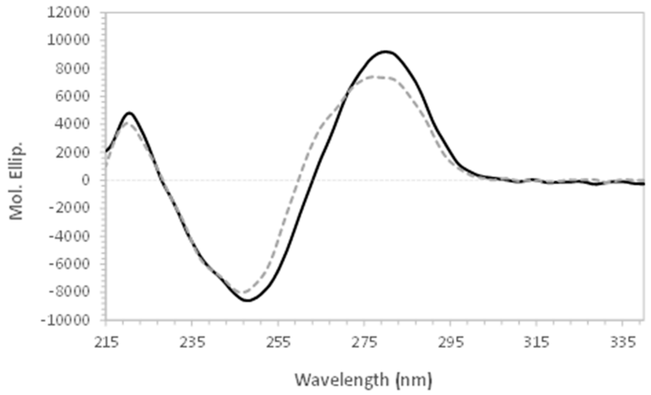

Figure S7. CD spectra of 10 μM substrate **18** (dashed grey line) or substrate **19** (bold black line) in Hepes-LiOH buffer (30 mM, pH 7.4) containing 2 mM $MgCl_2$, 50 mM NH_4Ac and 100 mM KCl.

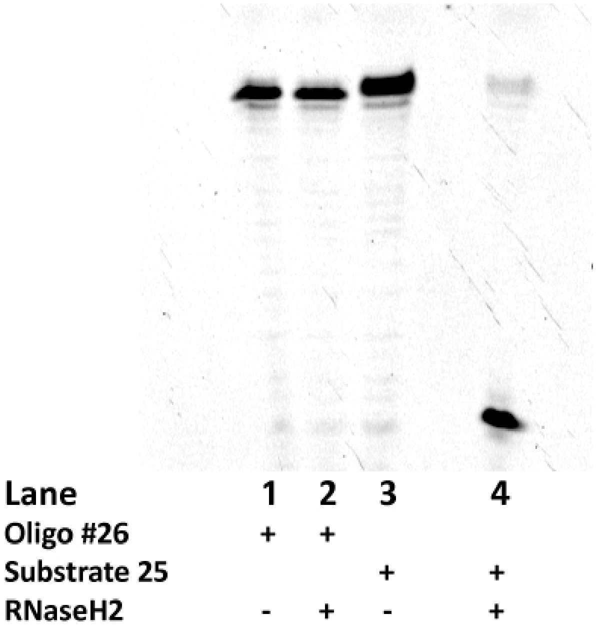

Figure S8. RNase H2 activity on single stranded DNA oligonucleotide with a single embedded ribonucleotide. Reactions were performed as described in Materials and Method for 10 minutes using 20 ng of single-stranded oligonucleotide #26 or double stranded substrates and 2 nM RNase H2.

Supplementary Bibliography

1. Del Villar-Guerra, R., Trent, J. O. & Chaires, J. B. (2018). G-Quadruplex Secondary Structure Obtained from Circular Dichroism Spectroscopy. 57, 7171-7175.
2. Kypr, J., Kejnovská, I., Renciuk, D. & Vorlícková, M. (2009). Circular dichroism and conformational polymorphism of DNA. *Nucleic Acids Res* 37, 1713-25.
3. Kharel, P., Becker, G., Tsvetkov, V. & Ivanov, P. (2020). Properties and biological impact of RNA G-quadruplexes: from order to turmoil and back. *Nucleic Acids Res* 48, 12534-12555.
4. Calabrese, D. R., Chen, X., Leon, E. C., Gaikwad, S. M., Phyo, Z., Hewitt, W. M., Alden, S., Hilimire, T. A., He, F., Michalowski, A. M., Simmons, J. K., Saunders, L. B., Zhang, S., Connors, D., Walters, K. J. & Mock, B. A. (2018). Chemical and structural studies provide a mechanistic basis for recognition of the MYC G-quadruplex. 9, 4229.
